# A Study on the Estimation of RIC (Radiation-Induced Conductivity) of Proton Irradiated Polyimide

**DOI:** 10.3390/polym15020337

**Published:** 2023-01-09

**Authors:** Yoshitaka Miyaji, Hiroaki Miyake, Yasuhiro Tanaka

**Affiliations:** 1 Advanced Technology R&D Center, Mitsubishi Electric Corp., Amagasaki, Hyogo 661-8661, Japan; 2 Department of Mechanics, Graduate School of Integrative Science and Engineering, Tokyo City University, Setagaya Campus, Setagaya, Tokyo 158-8557, Japan

**Keywords:** insulation materials, polyimide, radiation, proton, RIC (radiation-induced conductivity)

## Abstract

The recent expansion of the application environment of power electronics to high-radiation environments will cause the deterioration of insulation materials used in power electronics due to charging caused by cosmic ray irradiation. The charging phenomena should induce malfunctions in power electronics. Therefore, it is important to understand the insulation characteristics of insulation materials irradiated with protons, electrons, etc., to improve the reliability of power electronics. With respect to the above, there are few reports on the RIC (radiation-induced conductivity) of insulation materials irradiated with proton beams. In this paper, we experimentally evaluated the RIC of PI (polyimide) films irradiated with proton beams under various irradiation conditions. We also studied a calculation method to estimate the measured RIC of the PI. As a result, we clarified that the total conductivity of the PI increased under non-penetrating irradiation conditions and saturated under penetrating irradiation conditions. The reason for this is that the higher the irradiation energy, the deeper the maximum proton penetration depth under non-penetrating irradiation conditions. On the other hand, the conductivity characteristics did not change under penetrating conditions because the penetration depth was the same as the sample thickness. We also developed a calculation method to estimate the conductivity of the entire PI irradiated with proton beams. The estimated data calculated by the above method were analytically fitted with the measured data for most irradiation energy conditions. It is suggested that the above calculation method can estimate the conductivity of the entire PI irradiated with proton beams, regardless of penetrating or non-penetrating irradiation, based on the relationship between the RIC and dose rate of the PI irradiated under penetrating conditions. In the future, we will incorporate the results of this study into a computational model of space charge accumulation inside insulation materials to verify the influence of the RIC caused by proton irradiation on space charge accumulation.

## 1. Introduction

In recent years, the improvements in the reliability of power electronics have triggered many studies related to electrical insulation design technologies [1,2,3,4]. In these studies, their common concern is that the expansion of the application environment of power electronics to high-altitude and/or high-radiation environments will cause the deterioration of insulation materials used in power electronics due to discharge in low-pressure environments and/or charging caused by cosmic ray irradiation [5,6,7]. With respect to high-radiation environments, it has been reported that the exposure of high-energy charged particles, such as protons, electrons, etc., to insulation materials gives rise to the charge accumulations in the insulation materials due to changes in the insulation characteristics of the insulation materials [3,4,8]. The charging phenomena should induce malfunctions in power electronics. In addition, dielectric breakdown due to the charging and/or deterioration of insulation materials will make power electronics inoperable.

From the above background, it is important to understand the accumulation characteristics of the space charge in insulation materials irradiated with high-energy charged particles to improve the reliability of power electronics. Therefore, it is desirable to clarify the mechanism based on the calculation of the space charge accumulation in insulation materials irradiated with high-energy charged particles in the future. As a first step in the development of the calculation method of the space charge accumulation, it is necessary to propose a calculation method for the RIC (radiation-induced conductivity) caused by irradiation with high-energy charged particles. With respect to the above, there are many reports on the RIC of insulation materials irradiated with electron beams [9,10,11,12,13,14], while there are few reports on insulation materials irradiated with proton beams [15,16,17,18]. In this paper, we report experimental and computational studies on the RIC of proton irradiated insulation materials. In particular, we propose a calculation method to estimate the conductivity of the insulation materials irradiated with proton beams, regardless of penetrating or non-penetrating irradiation, based on the relationship between the RIC and dose rate of the insulation materials irradiated at penetrating conditions.

## 2. Materials and Measurement Methods

**Figure 1:**
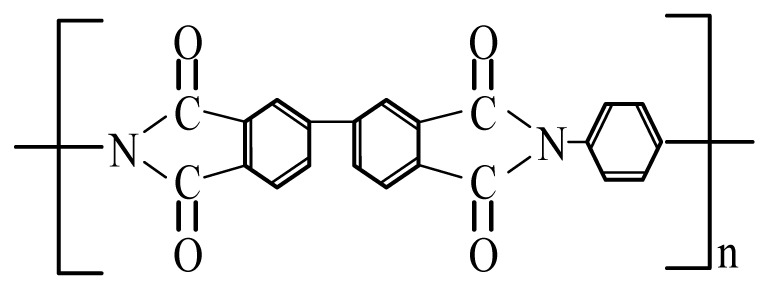
Chemical structure of PI.

Test samples were commercially available PI (polyimide) films (50 μm thickness) whose chemical structure is shown in Figure 1 [15,16,17]. To simulate the deterioration of the PI in high-radiation environments, the samples were irradiated with proton beams using a 3 MeV tandem accelerator facility at QST (Takasaki Advanced Radiation Research Institute of National Institute for Quantum and Radiation Science and Technology, Gunma, Japan) [19]. The following were the irradiation conditions: the irradiation energy was 1.0–2.5 MeV, the irradiation current density was 30 nA/cm^2^, the irradiation time was 30 min, and the vacuum pressure during proton irradiation was 10^−4^–10^−5^ Pa. Table 1 shows the maximum proton penetration depth in the samples under the above irradiation conditions calculated using SRIM (Stopping and range of ions in matter) [20].

**Figure 2:**
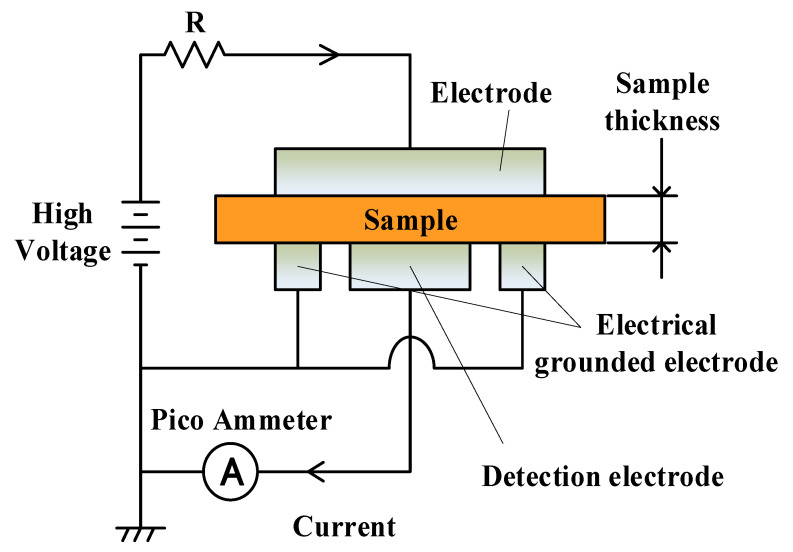
A principle of the ASTM method.

The conductivity of the samples was measured using the ASTM (American society for testing and materials) method [15,16,21]. Figure 2 shows a principle of the ASTM method. The samples were sandwiched between the high voltage electrode and the detection electrode with the guard electrode. The following were the conductivity measurement conditions: a DC electric field of 100 kV/mm was applied to the samples, and the maximum measurement time was 180 min. Since applying the electric field to the bulks of the samples causes conduction currents to flow through them, the conductivity, *σ*, was calculated with Equation (1).

(1) σ=J/E

In the equation, *J* is the measured current density and *E* is the electric field applied to the samples. The nomenclature for the parameters is listed in Table A1.

## 3. Measurement Results

**Figure 3:**
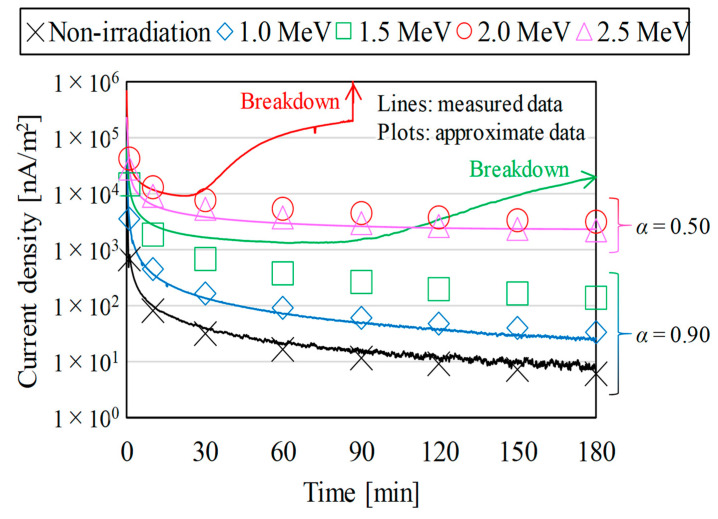
The irradiation energy dependence of the current density through the bulks of the proton irradiated samples after 1 day of irradiation.

**Figure 4:**
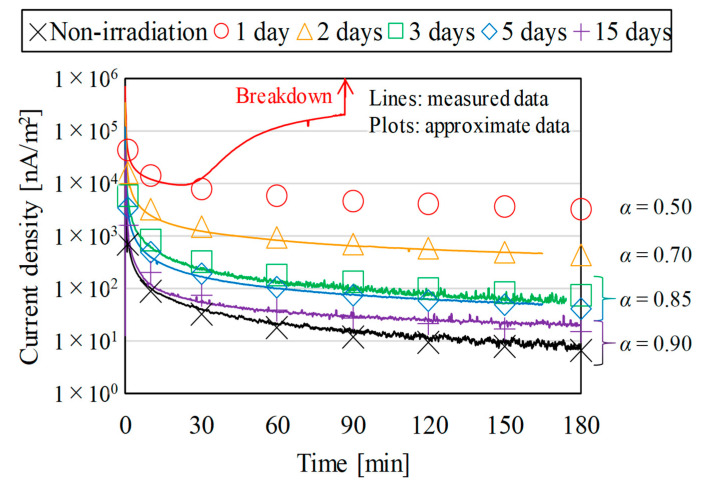
The change over time of the current density through the bulks of the proton irradiated samples under the irradiation energy condition of 2.0 MeV.

Figure 3 and Figure 4 show the measured results of the current density through the bulks of the proton irradiated samples as time progressed. The lines show the measured data [15,16] and the plots show the approximate data estimated by using the Maxwell–Wagner theory of composite layers [22,23], which, based on the measured data, can be expressed as a function of time with Equation (2).

(2) $$J=J_{\left(t=1\right)}\cdot t^{-\alpha }$$

In the equation, *J*_(*t* = 1)_ is *J* at *t =* 1, *t* is the elapsed time after the start of measurements, and *α* is a constant. The nomenclature for the parameters is listed in Table A1.

In Figure 3, *α* in Equation (2) was set to 0.90 or 0.50 for the non-penetrating or penetrating irradiated samples, respectively. The figure shows that the RIC caused by proton irradiation would make the samples highly conductive. The main factors that cause RIC are the activation of the samples, the generation of vacancies and the ionization in the samples due to the cleavage of molecular chains, and the application of irradiation energy to the samples.

In Figure 4, *α* in Equation (2) was changed from 0.50 to 0.90 with the elapsed time after proton irradiation. The figure shows that the conductivity of the proton irradiated samples reverted to that of the non-irradiated samples as time passed after irradiation. The phenomenon is so-called DRIC (delayed RIC) [24,25]. DRIC is defined as the phenomenon of the bulk of a sample remaining highly conductive even after irradiation. The recombination of electron-hole pairs generated inside a sample over time should suppress the generation of polarized charge and cause DRIC. Note that the approximate data for the irradiation energy conditions of 1.5–2.0 MeV in Figure 3 and the elapsed time conditions after 1 day of proton irradiation in Figure 4 will be used in the following discussions because the samples broke down during the measurements.

## 4. Calculation Methods

The RIC caused by electron irradiation can be expressed as a function of the dose rate with Equation (3) [9,24,25].

(3) $$\sigma_{\mathrm{RIC}}=k_{\mathrm{RIC}}{\left(\frac{dD}{dt}\right)}^{\Delta }$$

In the equation, *σ*_RIC_ is the RIC, *dD/dt* is the dose rate, and *k*_RIC_ and Δ are constants. Based on this relation, we will estimate the conductivity, including the RIC, of the entire sample irradiated with proton beams.

**Figure 5:**
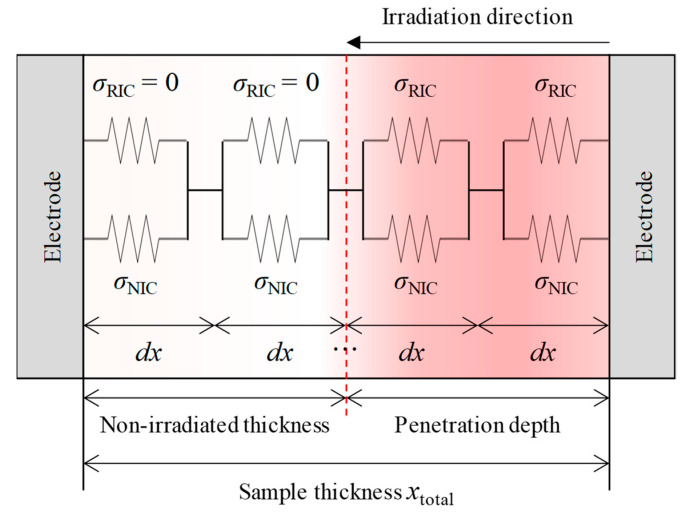
An equivalent circuit of the conductivity of the entire sample irradiated with proton beams.

Figure 5 shows an equivalent circuit of the conductivity, including the RIC, of the entire sample. The right side of the figure indicates the irradiated surface of the sample, and the left side indicates the non-irradiated surface. The total conductivity, *σ*_total_, of the entire sample can be expressed as a function of the intrinsic conductivity of the non-irradiated samples and the RIC with Equations (4) or (5). Incidentally, the total conductivity, *σ*_total_, is given as the harmonic mean of the sum of the intrinsic conductivity and the RIC of micro-intervals *dx* with equal weights, as in Equation (4).

(4) $$\sigma_{\mathrm{total}}={\left[\int {\left(\sigma_{\mathrm{NIC}}+\sigma_{\mathrm{RIC}}\right)}^{-1}dx\right]}^{-1}\cdot x_{\mathrm{total}}$$

In the equation, *σ*_NIC_ is the NIC (non-irradiated conductivity) of the non-irradiation samples and *x*_total_ is the total thickness of the samples. In addition, discretizing the above equation with the substitution of Equation (3) yields Equation (5).

(5) $$\sigma_{\mathrm{total}}={\left[\sum {\left\{\sigma_{\mathrm{NIC}}+k_{\mathrm{RIC}}{\left(\frac{dD}{dt}\right)}^{\Delta }\right\}}^{-1}\right]}^{-1}\cdot \frac{x_{\mathrm{total}}}{\Delta x}$$

In the equation, Δ*x* is a micro-interval with finite size. Furthermore, replacing *k*_RIC_ in the above equation with *k*_DRIC_, which can be expressed as a function of time with Equation (6) [10,25,26], reflects the influence of the DRIC on the total conductivity.

(6) $$k_{\mathrm{DRIC}}=\frac{k_{\mathrm{RIC}}}{1+\left(t^{\prime }-t_{0}^{\prime }\right)/\tau }$$

**Figure 6:**
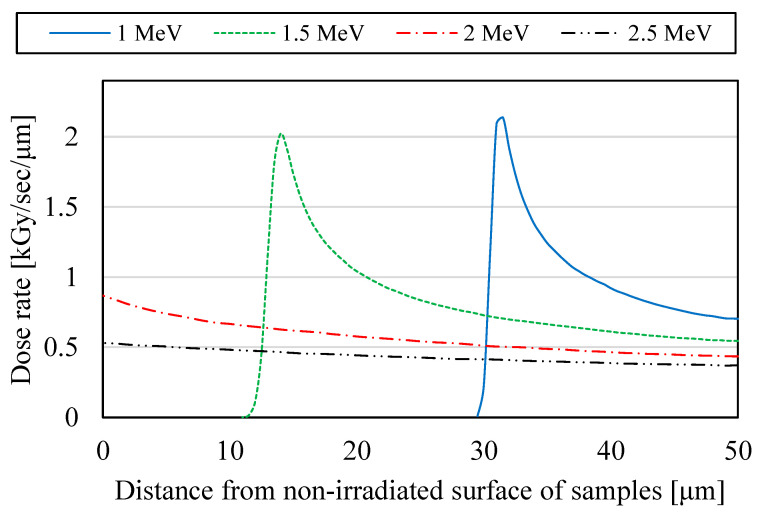
The dose rate distributions of the proton irradiated PI samples calculated using TRIM.

In the equation, $t^{\prime }$ is the elapsed time after proton irradiation, $t_{0}^{\prime }$ is $t^{\prime }$ at which the conductivity data were measured as the basis for DRIC evaluations, and *τ* is the time constant of the DRIC. Figure 6 shows the dose rate distributions of the proton irradiated samples calculated with Equation (7) [9,26].

(7) $$\frac{dD}{dt}=j_{\mathrm{irrad}}\cdot \frac{E_{\mathrm{deposit}}}{\rho \Delta x}$$

In the equation, *j*_irrad_ is the irradiation current density, *E*_deposit_ is the deposit energy per unit length of proton beams to samples calculated using TRIM (Transport of ions matter) [20], and *ρ* is the density of the samples. The nomenclature for the parameters is listed in Table A1. In Figure 6, incidentally, the irradiation energy is absorbed in the samples under non-penetrating irradiation conditions of 1.0–1.5 MeV. On the other hand, it is relatively difficult for irradiation energy to be absorbed in the samples under penetrating irradiation conditions of 2.0–2.5 MeV.

## 5. Calculation Results and Comparison with Measurement Results

**Figure 7:**
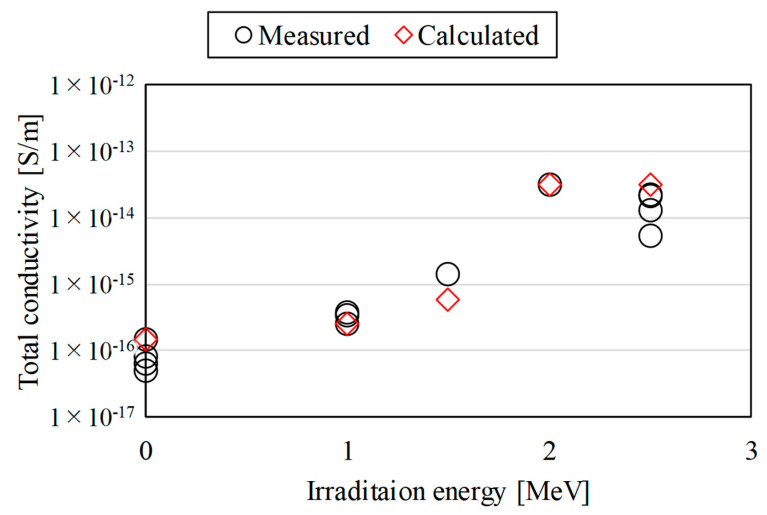
Calculated results of the total conductivity of the proton irradiated samples at 180 min after the start of the measurements. The irradiation energy dependence of conductivity after 1 day of proton irradiation.

**Figure 8:**
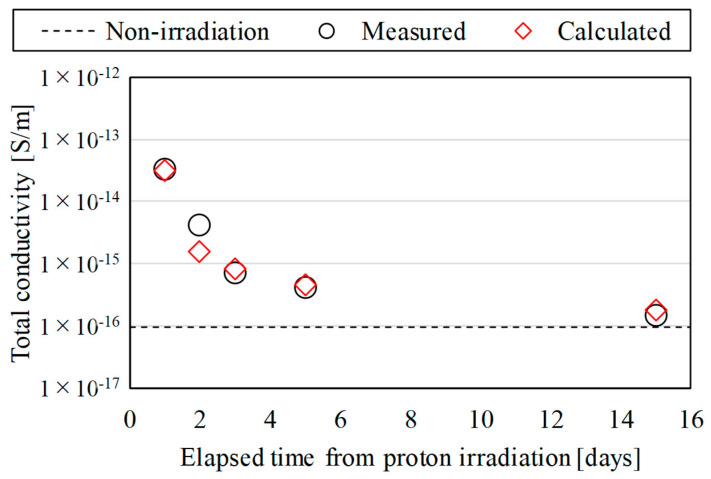
Calculated results of the total conductivity of the proton irradiated samples at 180 min after the start of the measurements. The change over time of conductivity under the irradiation energy condition of 2.0 MeV.

Figure 7 and Figure 8 show the estimated results of the total conductivity of the proton irradiated samples. The black circle plots are the measured data based on Equation (1) and Figure 3. The red diamond-shaped plots are the estimated data based on Equations (5) and (6) and Figure 6.

In Figure 7, *k*_RIC_ and Δ in Equation (5) were set to 2.0 × 10^−16^ and 0.80 [10,12], respectively. The measured data in the figure show that the total conductivity of the samples increased in the irradiation energy range of 1.0–2.0 MeV and saturated in the irradiation energy range of 2.0–2.5 MeV. The reason for this is that the irradiation energy range of 1.0–2.0 MeV corresponded to non-penetrating conditions, as shown in Table 1, and the higher the irradiation energy, the deeper the maximum proton penetration depth. On the other hand, the irradiation energy range of 2.0–2.5 MeV corresponded to penetrating conditions, as shown in Table 1, and the conductivity characteristics did not change because the penetration depth was the same as the sample thickness. Figure 7 shows that the estimated data were analytically fitted with the measured data for most irradiation energy conditions. Based on Equation (3), this result suggests that setting *k*_RIC_ and Δ appropriately can estimate the conductivity of the entire PI irradiated with proton beams regardless of penetrating or non-penetrating irradiation.

In Figure 8, *k*_RIC_ and Δ in Equation (5) were set to 2.0 × 10^−16^ and 0.80, respectively, as in Figure 7, and *τ* in Equation (6) was set to 0.05 [9]. The measured data in the figure show that the conductivity of the proton irradiated samples reverted to that of the non-irradiated samples as time passed after irradiation. The reason for this is that the recombination of electron-hole pairs generated inside the samples over time should suppress the generation of polarized charge, as shown in Section 3. Figure 8 shows that the estimated data were analytically fitted with the measured data for most of the elapsed time after proton irradiation. This result supports the suggestion in Figure 7.

## 6. Conclusions

We experimentally evaluated the RIC of PI films irradiated with proton beams under various irradiation conditions. We also studied a calculation method to estimate the measured RIC of the PI. As a result, we clarified that the total conductivity of the PI increased under non-penetrating irradiation conditions and saturated under penetrating irradiation conditions. The reason for this is that the higher the irradiation energy, the deeper the maximum proton penetration depth under non-penetrating irradiation conditions. On the other hand, the conductivity characteristics did not change under penetrating conditions because the penetration depth was the same as the sample thickness. We also developed a calculation method to estimate the conductivity of the entire PI irradiated with proton beams. The estimated data calculated by the above method were analytically fitted with the measured data for most irradiation energy conditions. Therefore, it is suggested that the above calculation method can estimate the conductivity of the entire PI irradiated with proton beams, regardless of penetrating or non-penetrating irradiation, based on the relationship between the RIC and dose rate of the PI irradiated under penetrating conditions. In the future, we will incorporate the results of this study into a computational model of space charge accumulation inside insulation materials [27,28] to verify the influence of RIC caused by proton irradiation on space charge accumulation. We will also assess the influence of RIC on electron-hole pair generation [10,29,30], the change in injection barriers on the irradiated surface of insulation materials [17,18], etc.

## Figures and Tables

**Table 1:** The maximum proton penetration depths in the PI samples calculated using SRIM.

Irradiation Energy Condition	Maximum Proton Penetration Depth	Type of Proton Irradiation
1.0 MeV	19 μm	Non-penetrating
1.5 MeV	37 μm	Non-penetrating
2.0 MeV	59 μm	Penetrating
2.5 MeV	84 μm	Penetrating

## Data Availability

The data presented in this study are available on request from the corresponding author.

## Appendix A

Table A1 shows the nomenclature for the parameters used in the equations in this paper. We hope that Table A1 will assist you in reading this paper.

**Table A1:** The nomenclature for the parameters used in the equations in this paper.

Symbol	Content	1st Appearance
*σ*	Conductivity	Equation (1)
*J*	Current density	same as above
*E*	Electric field	same as above
*J*_(*t* = 1)_	*J* at *t* = 1	Equation (2)
*t*	Elapsed time after the start of the ASTM measurements	same as above
*α*	Constant for Maxwell-Wagner’s theory of composite layers	same as above
*σ*_RIC_	RIC (radiation-induced conductivity)	Equation (3)
*dD/dt*	Dose rate	same as above
*k*_RIC_*,* Δ	Constants for the relationship between the RIC and dose rate	same as above
*σ*_total_	Total conductivity of the entire sample	Equation (4)
*σ*_NIC_	NIC (non-irradiated conductivity)	same as above
*dx*	Micro-intervals along the thickness of the samples	same as above
*x*_total_	Total thickness of the samples	same as above
Δ*x*	Micro-interval with finite size along the thickness of the samples	Equation (5)
*k*_DRIC_	Constant for the DRIC (delayed RIC)	Equation (6)
*t’*	Elapsed time after proton irradiation	same as above
*t’*_0_	*t* at which the conductivity data were measured as the basis forDRIC evaluations	same as above
*τ*	Time constant of the DRIC	same as above
*j*_irrad_	Irradiation current density	Equation (7)
*E*_deposit_	Deposit energy per unit length of proton beams to samples calculated using TRIM (transport of ions matter)	same as above
*ρ*	Density of the samples	same as above

## References

[1] He J., Zhang D., Torrey D. Recent advances of power electronics applications in more electric aircrafts. Proceedings of the 2018 AIAA/IEEE Electric Aircraft Technologies Symposium.

[2] Nakashima J., Horiguchi T., Mukunoki U., Hatori K., Tsuda R., Uemura H., Hagiwara M., Urakabe T. (2022). Investigation of full SiC power modules for more electric aircraft with focus on FIT rate and high-frequency switching. IEEE Trans. Ind. Appl..

[3] Miyaji Y., Ishikawa H., Tajiri K., Shiota H., Enoki K., Miyake H., Tanaka Y. Study on charge distributions in irradiated insulation materials of power electronics. Proceedings of the 2020 International Symposium on Electrical Insulating Materials (ISEIM).

[4] Enoki K., Miyake H., Tanaka Y., Miyaji Y., Ishikawa H., Tajiri K., Shiota H. Measurements of charge distributions in insulation materials of power electronics equipment irradiated by proton/electron. Proceedings of the 2020 IEEE Conference on Electrical Insulation and Dielectric Phenomena (CEIDP).

[5] Abadie C., Billard T., Lebey T. Influence of pressure on partial discharge spectra. Proceedings of the 2016 IEEE Electrical Insulation Conference (EIC).

[6] (1987). NCRP Report 94.

[7] Sato T., Yasuda H., Niita K., Endo A., Sihver L. (2008). Development of PARMA: PHTIS-based analytical radiation model in the atmosphere. Radiat. Res..

[8] Miyake H., Tanaka Y., Diaham S. (2021). Space charge accumulation phenomena in PI under various practicable environment. Polyimide for Electronic and Electrical Engineering Applications.

[9] Yang G.M., Sessler G.M. (1992). Radiation-induced conductivity in electron- beam irradiated insulating polymer films. IEEE Trans. Eletcr. Insul..

[10] Sessler G.M., Figueiredo M.T., Ferreira G.F.L. (2004). Models of charge transport in electron-beam irradiated insulators. IEEE Trans. Dielectr. Electr. Insul..

[11] Levy L., Paulmier T., Dirassen B., Inguimbert C., Eesbeek M.V. (2008). Aging and prompt effects on space material properties. IEEE Plasma Sci..

[12] Paulmier T., Dirassen B., Payan D., Eesbeek M.V. (2009). Material charging in space environment: Experimental test simulation and induced conductive mechanisms. IEEE Trans. Dielectr. Electr. Insul..

[13] Li G., Li S., Pan S., Min D. (2016). Dynamic charge transport characteristics in polyimide surface and surface layer under low-energy electron radiation. IEEE Trans. Dielectr. Electr. Insul..

[14] Pan S., Min D., Wang X., Hou X., Wang L., Li S. (2019). Effect of electron irradiation and operating voltage on the deep dielectric charging characteristics of polyimide. IEEE Trans. Nucl. Sci..

[15] Uchiyama R., Hara T., Homme Y., Miyake H., Tanaka Y. Degradation phenomena of electric property on polymeric materials films irradiated by proton. Proceedings of the 2012 Annual Report Conference on Electrical Insulation and Dielectric Phenomena.

[16] Uchiyama R., Horiguchi K., Wang Z., Miyake H., Tanaka Y. Evaluation for insulation degradation properties in proton beam irradiated polyimide films. Proceedings of the 9th Spacecraft Environment Symposium.

[17] Miyake H., Uchiyama R., Tanaka Y. The relationship between charge accumulation and scission of molecular chain in the proton irradiated PI. Proceedings of the 2016 IEEE International Conference on Dielectrics (ICD).

[18] Sato N., Suzuki K., Chiba U., Miyake H., Tanaka Y., Okumura T., Kawakita S., Takahashi M., Koga K. Photoelectron emission on polyimide films irradiated by proton. Proceedings of the 2018 Condition Monitoring and Diagnosis (CMD).

[19] Miyake H., Yoshida S., Mori T., Chiba U., Tanaka Y. Physicochemical analysis for fluorinated polymer films irradiated by proton. Proceedings of the 2017 IEEE Conference on Electrical Insulation and Dielectric Phenomenon (CEIDP).

[20] Ziegler J.F., Biersack J.P., Littmark U., Ziegler J.F., Biersack J.P., Littmark U. (1985). The stopping and range of ions in solids. Stopping and Ranges of Ions in Matter.

[21] (2012). Standard Test Methods for DC Resistance or Conductance of Insulating Materials.

[22] Okazaki K. (1993). Dielectric absorption current of ferroelectric ceramics and a new theory based on space charge diffusion. Mem. Shonan Inst. Technol..

[23] Wagner K.W. (1914). Erklärung der dielektrischen Nachwirkungsvorgänge auf Grund Maxwellscher Vorstellungen. Arch. Electr. Eng..

[24] Wintle H.J. (1991). Models for the decay of radiation-induced conduction. IEEE Trans. Electr. Insul..

[25] Berkley D.A. (1979). Computer simulation of charge dynamics in electron-irradiated polymer foils. J. Appl. Phys..

[26] Gross B., Sessler G.M., West J.E. (1974). Charge dynamics for electron-irradiated polymer-foil electrets. J. Appl. Phys..

[27] Roy S.L., Teyssédre G., Laurent C., Dissado L.A., Montanari G.C. Relative importance of trapping and extraction in the simulation of space charge distribution in polymeric insulators under DC potentials. Proceedings of the 2007 IEEE International Conference on Solid Dielectrics.

[28] Miyaji Y., Ishikawa H., Shiota H., Tajiri K., Enoki K., Endo K., Miyake H., Tanaka Y. Study on deterioration of insulation materials used in power electronics irradiated by proton. Proceedings of the 2021 Symposium on Electrical and Electronic Insulating Materials and Applications in Systems.

[29] Roy S.L., Baudoin F., Griseri V., Laurent C., Teyssédre G. (2012). Space charge modeling in electron-beam irradiated polyethylene: Fitting model and experiments. J. Appl. Phys..

[30] Banda M.E., Roy S.L., Griseri V., Teyssédre G. (2020). Bipolar charge transport model to estimate charging and degradation processes in electron-beam irradiated LDPE films. J. Phys. D Appl. Phys..

